# Aphid Infestation and Predator Dynamics in Cultivated *Ruta chalepensis*: Evidence of *Myzus persicae* Adaptation and Natural Enemy Responses

**DOI:** 10.3390/insects16111088

**Published:** 2025-10-24

**Authors:** Eugênio E. Oliveira, Tarciza F. Nascimento, Leonardo S. Francesco, Thiago Svacina, César León, Carlos N. Gomes, Luis O. Viteri Jumbo, Maria José González Armijos, Karina S. Vilca Mallqui, Guy Smagghe

**Affiliations:** 1 Programa de Pós-Graduação em Biotecnologia, Universidade Federal do Tocantins, Gurupi 77410-530, TO, Brazilluis.viteri@uft.edu.br (L.O.V.J.); 2 Departamento de Entomologia, Universidade Federal de Viçosa, Viçosa 36570-900, MG, Brazil; 3 Departamento de Agronomia, Universidade Federal de Viçosa, Viçosa 36570-900, MG, Brazil; 4 Programa de Pós-Graduação em Produção Vegetal, Universidade Federal do Tocantins, Gurupi 77410-530, TO, Brazil; 5 Departamento de Bioquímica e Imunologia, Instituto de Ciências Biológicas, Universidade Federal de Minas Gerais, Av. Antônio Carlos, 6627, Belo Horizonte 31270-901, MG, Brazil; 6 Academic Department of Agronomy, National University Santiago Antunez de Mayolo, Huaraz 02002, Ancash, Peru; 7 Institute Entomology, Guizhou University, Guiyang 550025, China; 8 Department of Biology, Vrije Universiteit Brussel (VUB), 1050 Brussels, Belgium

**Keywords:** rue herbs, insect repellent plants, aphids, non-conventional crops

## Abstract

**Simple Summary:**

*Ruta chalepensis*, commonly known as rue, is a medicinal plant traditionally known for its insect-repellent properties. However, little is known about which insect species can successfully feed on or infest this plant. In this study, we report, for the first time, the presence of the aphid *Myzus persicae* (green peach aphid) infesting rue plants under Neotropical conditions. Here, we demonstrate *M. persicae* infestations occurring in agricultural fields in the Brazilian state of Minas Gerais. Plant and insect species were accurately identified using herbarium records and microscopic analysis. These findings highlight the remarkable adaptability of *M. persicae* and show how plant domestication and monoculture practices can increase pest pressure, even in crops traditionally believed to resist insect pests.

**Abstract:**

*Ruta chalepensis* (Rutaceae), commonly known as rue, is traditionally recognized for its medicinal applications and cultural associations with mysticism. In addition to its ethnobotanical relevance, *R. chalepensis* has been reported to possess insect-repellent and pesticidal properties. However, domestication and the adoption of monoculture practices can compromise inherent plant defenses, potentially increasing susceptibility to herbivore pressure. In this context, we report, for the first time, the occurrence of infestations by the highly polyphagous aphid *Myzus persicae* (Sulzer) (Hemiptera: Aphididae) on *R. chalepensis* cultivated under Neotropical conditions. The infestations were documented in commercial rue fields located in the Zona da Mata region, Minas Gerais, Brazil, in two consecutive years. Plant material was taxonomically verified and deposited in the VIC Herbarium at the Universidade Federal de Viçosa. Aphid specimens, including both apterous and alate morphs, were examined under a stereomicroscope to confirm species identity. Field sampling revealed a notable interaction between *M. persicae* and native natural enemies, particularly predatory spiders. Aphid density per plant declined markedly from 14.1 to 6.2 individuals, coinciding with an observed increase in spider activity. These findings expand our understanding of *M. persicae*’s ecological plasticity and highlight the vulnerability of non-conventional crops to generalist pests. They also underscore the importance of implementing targeted pest surveillance and integrated pest management (IPM) approaches in underutilized or alternative cropping systems.

## 1. Introduction

The Rutaceae family comprises a diverse group of aromatic plants distributed across most temperate and tropical regions, primarily as shrubs and small trees [1]. Members of this family are chemically rich, producing bioactive compounds such as furanocoumarins, furano alkaloids, acridone alkaloids, phenolics, and terpenes, which contribute to their traditional medicinal use and known insecticidal properties [2]. Among these, *Ruta* species, particularly *R. graveolens*, *R. corsica*, and *R. chalepensis*, have a long-standing history of use in ethnomedicine and agriculture [3]. In the Neotropical region, the growing demand for bioactive products of rue plants has promoted social development among small-scale farmers, which has led to the reports of yield losses due to phytopathogens [4]. *Ruta chalepensis* is not unlike the majority of *Rutaceae* plants, with numerous compounds present in its parts, including leaves, stems, roots, flowers, and seeds [5]. In particular, is valued for its distinct fragrance, therapeutic potential (e.g., antipyretic, antihypertensive, and antimicrobial effects), and ecological resilience [6,7,8]. Its phytochemical complexity includes organ-specific accumulations of flavonoids (notably rutin), terpenoids, and phenolic acids, with recent studies identifying genetic elements involved in flavonoid biosynthesis [5,9,10]. These features suggest biotechnological potential, especially for producing natural bioactive compounds with pesticidal functions [8]. Due to its low-input cultivation and presumed pest resistance, *R. chalepensis* is increasingly cultivated as an ornamental and medicinal crop. While numerous laboratory and field studies have demonstrated insect-repellent and toxic effects of rue extracts on various insect pests [11], there is a striking lack of documentation on actual pest infestations in cultivated *R. chalepensis*. This absence has been largely attributed to the plant’s secondary metabolites [12]. Field studies from regions such as Taif, Saudi Arabia, reported no aphid or insect pest presence on rue plants over multiple seasons [13]. Even though aphid infestations have been recorded in other Rutaceae species, no confirmed reports exist for *R. chalepensis* or any *Ruta* spp., reinforcing assumptions of its exceptional pest resistance [14,15].

Here, we present the first field report of *Myzus persicae* (Sulzer) (Hemiptera: Aphididae), which is a highly polyphagous and economically significant pest, colonizing *R. chalepensis* under Neotropical conditions. This finding challenges the long-held perception of rue’s pest immunity and opens new lines of inquiry into its vulnerability under domestication. Our work highlights the need to re-evaluate insect–plant interactions in traditionally “resistant” medicinal species and provides foundational data for developing sustainable phytosanitary and crop management strategies in *R. chalepensis* cultivation systems.

## 2. Material and Methods

### 2.1. Study Place

**Figure 1:**
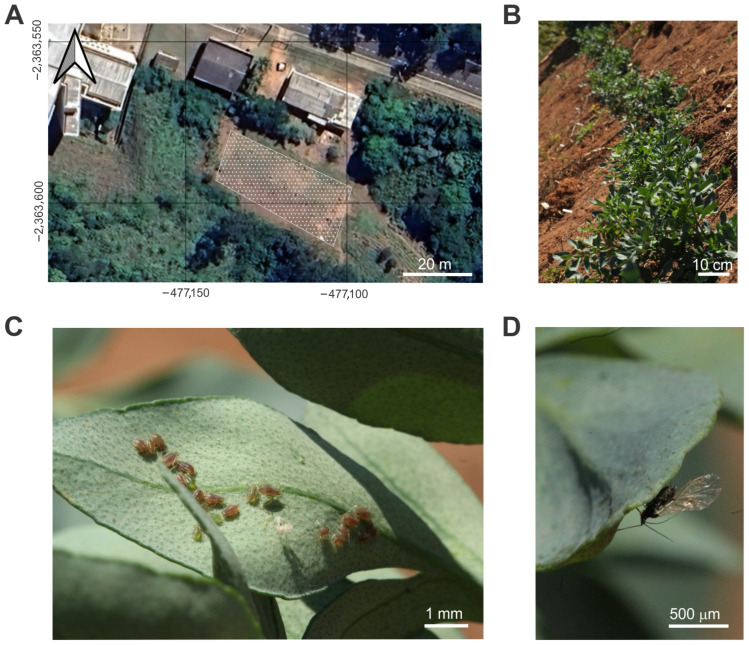
Study site in Viçosa, Minas Gerais State, Brazil. (**A**) Geospatial mapping of the *R. chalepensis* cultivation field. (**B**) *R. chalepensis* plants at the time of insect sampling. (**C**) Wingless *Myzus persicae* individuals feeding on the abaxial surface of *R. chalepensis* leaves. (**D**) Winged *M. persicae* initiating infestation on a previously uninfested *R. chalepensis* plant.

We established *R. chalepensis* fields in two consecutive years by planting rue seedlings. The field located in an experimental field at the Federal University of Viçosa (UFV), located at coordinates 20°45′45.46″ S, 42°51′34.74″ W (Figure 1A). Firstly, on 27–28 April, and 5 May 2024, between 7:00 a.m. and 9:30 a.m., we planted a total of 189 seedlings (average height: 15.0 ± 1.5 cm). These seedlings were distributed across an area of approximately 630 m^2^, in planting holes measuring 24 cm in depth and 24 cm in diameter. The holes were prepared 15 days in advance using a 1:1 mixture of soil and bovine manure.

According to soil analysis, the field had a pH of 5.5, an effective cation exchange capacity (CTC_ee_f) of 6.68 cmolc/dm^3^, a base saturation (V%) of 24.3%, a remaining phosphorus (P-rem) content of 25.9 mg/L, and a potential acidity (H^+^ + Al^3+^) of 4.95 cmolc/dm^3^. Seedlings were monitored daily and irrigated each morning, except on rainy days or when soil moisture was adequate, to maintain optimal water balance for successful establishment (Figure 1B). After the harvest of rue plants in September 2024, the field remained fallow until November 2024, when it was cultivated with lablab beans (*Lablab purpureus* (L.) Sweet) as a cover crop to prevent soil erosion, protect the soil, and control weeds. In March 2025, before seed production occurred, the lablab plants were mowed, and the biomass was left in the field to decompose. On 22 May 2025, we planted 148 rue seedlings (average height: 15.2 ± 1.3 cm) and monitored the occurrence of aphids and spiders. The first evaluation was conducted on 31 May 2025, followed by three additional evaluations on 28 June, 26 July, and 9 August 2025. Hole-opening procedures followed the same protocol described above; however, we applied two soil fertilization treatments: (1) a 1:1 mixture of soil and bovine manure, and (2) the same mixture supplemented with 80 g of mineral fertilizer (20-5-20, N–P–K, Eurochem Fertilizantes, Manhuaçu, MG, Brazil), aiming to increase leaf mass production.

### 2.2. Arthropod Sampling

The study site had previously been cultivated with the silkworm mulberry, *Morus alba* L., under insecticide-free conditions for over 30 years (Figure 1A). In the first year of cultivation, we recorded a notable aphid infestation, whereas in the second year, only a less intense infestation was observed on the *R. chalepensis* plants (Figure 1B–D). To monitor aphid population dynamics, we followed a sampling protocol adapted from previous studies [16,17]. Briefly, the experimental area was divided into plots, and sampling was conducted using an “N”-shaped walking pattern to ensure randomization and representative coverage. For the first year cultivation, we selected a total of 30 plants. Each plant was shaken three times over a large tray, and both aphids and spiders as biological control agents, were manually counted. Similar procedures were conducted in the second year of cultivation, using 30 plants per soil-fertilization treatment.

### 2.3. Plant Identification

In Brazil, rue seedlings are commonly sold as “female” (narrow-leaved) and “male” (broad-leaved) types. To ensure accurate species identification, we cultivated the seedlings until the flowering stage and collected various plant parts (e.g., leaves, fruits, and branches). These materials were taxonomically identified by specialists, and a voucher specimen was deposited in the VIC Herbarium of the Department of Plant Biology at the Federal University of Viçosa (UFV) under the accession number VIC 56924.

### 2.4. Aphid Identification

**Figure 2:**
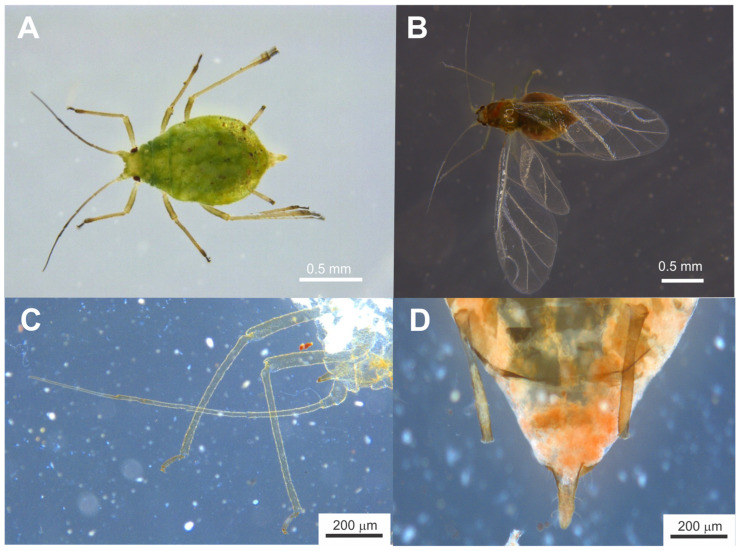
(**A**) Wingless *Myzus persicae* adult immediately after sampling from a *R. chalepensis* plant. (**B**) Winged *M. persicae* adult immediately after sampling from a *R. chalepensis* plant. (**C**) Microscopic image showing morphological details of the clarified aphid antennae. (**D**) Microscopic image showing morphological details of the clarified aphid caudal region.

Both winged and wingless morphotypes of aphids were collected during the infestation (Figure 2A,B) from various parts of the plant (e.g., leaves, stems, twigs) to confirm that the population was well established in the crop. Specimens were initially examined using a stereomicroscope (M205C, Leica Microsystems, Wetzlar, Germany), allowing high-resolution observation of key morphological features. Prior to final identification, samples were processed using a clearing protocol involving immersion in 10% (*w*/*v*) potassium hydroxide (KOH) solution for 12 h. Species identification was subsequently confirmed by a specialist from the National University Santiago Antúnez de Mayolo (Peru), using internationally recognized aphid identification keys [15].

## 3. Results

### 3.1. Identification of Plant Species

All rue plants were identified as *R. chalepensis* based on morphological characteristics, including leaflet shape, flower structure, and glandular dot patterns on the leaves, following standard botanical keys [18]. Field-collected specimens were compared with herbarium material for confirmation. The identification was validated by a plant taxonomy specialist, and a voucher specimen was subsequently deposited (VIC 56924) in the VIC Herbarium of the Federal University of Viçosa (UFV).

### 3.2. Identification of Aphid Species

The aphid species infesting *R. chalepensis* plants was identified as the green peach aphid, *M. persicae*, based on morphological characteristics and supported by references [15,19,20,21].

*Description of clarified wingless specimens* (Figure 2A): Head with long antennal tubercles bearing rounded excrescences on the inner faces, appearing convergent. Frontal setae are obtuse, up to 30 µm in length; those on the antennal tubercles are shorter. Antennae measure 0.65–0.80 times the body length; the terminal process is approximately 3–5 times longer than the basal part of antennal segment VI.

Antennal setae measure 0.16–0.33 times the basal diameter of antennal segment III, and antennal segments III–V lack secondary rhinaria (Figure 2C). The rostrum reaches the mid-thigh; the last rostral segment is 0.95–1.15 times longer than segment II of the hind tarsus and bears 2–4 additional setae. The thorax and legs are covered with short setae; apical tibial setae do not exceed their diameter. The first tarsal segments bear 3, 3, and 2 setae, respectively. The abdomen has a membranous dorsum with very short or imperceptible dorsal setae except on distal segments. Ventral setae are acute and 15–20 µm in length. Tergite VIII bears four ventral-like setae (Figure 2D). Siphunculi measure 0.20–0.28 times the body length, are clear but may have a dark apex, are without setae, and are weakly imbricated (Figure 2D). The marginal ridge is well developed with a slight medial constriction. The siphunculi length is approximately 4.75 to 7 times its width, exceeding the length of the cauda. The cauda is clear to dark, elongated, more than twice as long as wide (0.37–0.50 times the length of the siphunculi), triangular in shape, with 5–7 setae. The genital plate usually has two setae at the anterior part and 12–17 short setae along the posterior margin.

*Description of clarified winged specimens* (Figure 2B): Head with antennae approximately as long as the body. Terminal process of antennal segment VI is 3–5 times the length of its base. Antennal segment III bears 7–16 secondary rhinaria, while segments IV and V have none. The last rostral segment measures 0.11–0.12 mm in length and carries 2–4 additional setae. Legs are covered with short setae, with apical tibial setae not exceeding their diameter. First tarsal segments have 3, 3, and 2 setae, respectively.

The abdomen dorsum features a distinctive sclerotized central plate extending from segment III to V or VI, often fused with some intersegmental plates and post-siphuncular sclerites. Large marginal sclerites are present, with pre-siphuncular sclerites quite small and post-siphuncular sclerites large, showing broad stripes on the apical segments. Siphunculi are swollen in the distal half, with the swollen region approximately 1.25 to 1.5 times wider than the narrowest proximal half; the proximal half is wrinkled and lacks constrictions, and the swollen area is slightly imbricated. The cauda is triangular, about 1.5 times longer than its basal width, bearing 5 to 6 setae (Figure 2D).

### 3.3. Sampling of Aphids and Predatory Spiders

**Figure 3:**
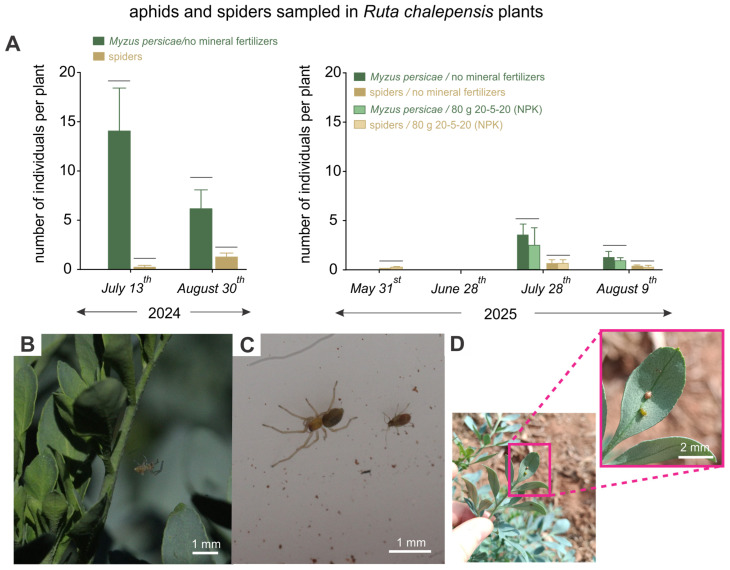
Aphid and predatory spider occurrence on *R. chalepensis* plants. (**A**) Average numbers of *M. persicae* and predatory spiders recorded on two sampling dates. (**B**) Predatory spider on its web on an *R. chalepensis* plant. (**C**) Predatory spider and aphid together inside a sampling tray. (**D**) Example of parasitized aphids recorded on the final sampling day of the 2025 season. (**A**,**B**) Bars aligned on the same horizontal line represent groups that are not significantly different, based on Tukey’s HSD test (*p* < 0.05).

The first sampling, conducted on 13 July 2024, recorded an average density of *M. persicae* of 14.07 ± 4.63 individuals per plant, which declined to 6.17 ± 1.52 by the second sampling on 10 August 2024 (Figure 3A). This reduction in aphid numbers coincided with an increase in predatory spider abundance, from 0.13 ± 0.08 to 1.20 ± 0.22 individuals per plant. Both taxa were frequently observed co-occurring on the same plants (Figure 3B,C). In the second rue season (2025), aphid and spider abundances did not differ significantly among fertilization treatments (Tukey’s HSD test; *p* > 0.05). Overall, infestations and spider occurrence were lower than in 2024 (Figure 3A). Aphids were detected only in the last two sampling periods, reaching 4.00 ± 0.92 and 1.10 ± 0.32 individuals per plant without mineral fertilization, and 3.40 ± 1.21 and 0.90 ± 0.24 with fertilization. Spiders were present in most periods, with densities ranging from 0.09 ± 0.03 to 0.04 ± 0.02 without fertilization, and from 0.07 ± 0.04 to 0.02 ± 0.02 with fertilization. Although no parasitoid wasps were recorded, parasitized aphids were found on some plants during the final sampling (Figure 3D).

## 4. Discussion

Here, we demonstrate for the first time that rue plants, specifically *R. chalepensis*, when cultivated as a monoculture, can be infested by aphids such as *M. persicae*. While morphological traits were sufficient for species-level identification, integrating molecular tools in future surveys would provide additional confirmation and help prevent misidentification in complex aphid groups. Furthermore, we documented the potential roll of naturally occurring predatory spiders in the dynamic fluctuations of these aphid populations. Although we could not identify the organisms parasitizing aphids in the second season, we recorded a lower intensity of aphid infestation alongside the presence of parasitized individuals. Our observations do not establish causality, and other factors (e.g., weather fluctuations or management of adjacent fields) cannot be excluded. Nevertheless, these findings are consistent with broader evidence of predator-mediated suppression of aphids in agroecosystems and highlight the relevance of characterizing local natural enemy assemblages.

The occurrence of *M. persicae* on *R. chalepensis* is unprecedented and raises questions about the adaptability of this generalist pest to plants traditionally considered chemically defended. Host plant selection by aphids is a sequential process involving multiple behavioral steps, where plants may be rejected based on physical and chemical cues at any stage [15,21,22]. Previous studies have highlighted key ecological traits of aphids, including rapid reproduction and sophisticated adaptation to host plant ecology, phenology, physiology, and biochemistry [23]. The aphid *M. persicae* is highly polyphagous, feeding on over 400 plant species across 40 botanical families, including Solanaceae, Brassicaceae, and Fabaceae [24]. However, no prior records exist of *M. persicae* infesting any *Ruta* species worldwide [13,14]. Research also shows that *M. persicae* efficiently discriminates among suitable host plants for its offspring, independent of phylogenetic proximity [25]. Future studies are needed to determine the geographic extent and frequency of *M. persicae* infestations in *R. chalepensis* and related *Ruta* species across different agroecological zones. Furthermore, while our findings provide valuable insights, it is important to note that they were derived from data collected over two consecutive years at a single site. Future investigations should, therefore, incorporate multi-site and multi-year datasets, along with a more detailed examination of environmental and agronomic variables, to enhance the generalizability and ecological relevance of the results.

It is also relevant to note that our findings rely on the morphological identification of *M. persicae*. While morphological traits have traditionally served as the foundation for aphid species identification, there is increasing recognition of the limitations inherent in relying solely on morphology, especially within species complexes [26,27,28]. Previous studies [29,30,31] have demonstrated that misidentification of aphid species can occur when exclusively using morphological keys, particularly in groups where diagnostic characters overlap or are influenced by environmental factors. Such potential misidentification is especially problematic in pest management contexts, where accurate species identification is essential for implementing effective control strategies. Therefore, integrating molecular identification methods (e.g., DNA barcoding or species-specific PCR) would strengthen the morphological characterization of *M. persicae* presented here and is recommended for future surveys.

Population declines during the season coincide with the presence of spiders and parasitized aphids, suggesting a role of natural enemies in moderating infestations. Spiders, as generalist predators, exhibit flexible foraging strategies that enable them to exploit aphids as prey throughout the crop’s phenological stages, particularly affecting early aphid population growth [24,32]. However, their predation on aphids is often biased toward winged individuals that become ensnared in webs, indicating that although spiders contribute to aphid suppression, their impact may be selective and influenced by aphid behavior and morphology [33]. Moreover, spider assemblages in agroecosystems are typically diverse, with different families and guilds occupying complementary feeding niches, thereby enhancing overall biological control through niche partitioning and temporal complementarity. Determining whether such predation measurably impacts aphid populations in rue requires further, broader investigations. Molecular gut-content analyses have confirmed spider predation on aphids throughout crop phenology, with families such as *Linyphiidae* notably impeding early aphid population growth [34,35]. While spider predation is supported, little is known about specific spider guild dynamics and prey preferences in *R. chalepensis* systems, which could optimize their conservation and deployment in IPM programs.

The presence of parasitized aphids suggests parasitoid wasp activity, although no adult parasitoids were recovered. Parasitoids typically exert strong top-down control on aphid populations by parasitizing immature stages, often resulting in delayed yet substantial reductions in aphid abundance. The complementary actions of parasitoids and spiders can create a multi-layered natural enemy complex that regulates aphid populations more effectively than either group alone. For example, whereas spiders may reduce aphid dispersal by preying on alates, parasitoids suppress population growth by reducing the number of reproductive individuals, together contributing to population stability.

Although fertilization can influence aphid population dynamics by altering plant nutritional quality, our data suggest that such bottom-up effects are context dependent and may be moderated by biotic interactions with natural enemies. Nitrogen-rich mineral fertilizers are generally known to increase phloem amino acid concentrations and enhance plant quality for *M. persicae*, potentially accelerating aphid population growth and supporting larger predator and parasitoid populations [36,37]. However, this effect was not evident in our 2025 data, suggesting that environmental conditions or biotic interactions may have moderated the expected fertilization-driven increase in aphid abundance. Although the soil conditions may have constrained optimal plant growth, *R. chalepensis* is rustic and tolerant of diverse soil types, including moderately acidic soils, particularly when supplemented with organic matter, as was performed in this study [23,38]. Further investigations are needed to elucidate predator guild dynamics, prey preferences, and temporal interactions between spiders, parasitoids, and aphids in this system. Molecular gut-content analysis and field-based exclusion experiments could provide deeper insights into predation rates and parasitoid efficacy throughout the crop life cycle, ultimately informing targeted biological conservation control strategies.

Despite *R. chalepensis*’ rich chemical arsenal, including flavonoids, alkaloids, terpenes, and phenolic compounds with insect-repellent and insecticidal properties [2,5], the successful establishment of *M. persicae* suggests the possible circumvention or breakdown of these defenses. This may be explained by the high ecological plasticity and adaptability of *M. persicae*, a generalist able to feed hundreds of species and overcome plant chemical defenses via enzymatic detoxification, rapid reproduction, and selective host discrimination [15,32,39]. Moreover, cultivation conditions, such as uniform plant age, reduced environmental stress, and absence of prior herbivory, could influence the biosynthesis or localization of secondary metabolites [8,9], creating temporal windows of vulnerability. Intraspecific variability and environmental modulation of key bioactive compounds like rutin, which fluctuate with developmental stages and environmental factors [10], must also be considered.

Although *R. chalepensis* has historically been considered resistant to aphid infestation [13,15,25], our findings demonstrate that, under certain cultivation regimes, even chemically defended plants can become susceptible to generalist pests such as *M. persicae* [20,39]. The successful colonization of chemically defended plants such as *R. chalepensis*, which is rich in furanocoumarins and alkaloids [40,41], by the generalist herbivore *M. persicae* can be attributed to its exceptional ecological plasticity. This adaptability is achieved through a two-pronged strategy involving biochemical detoxification and specialized feeding behavior [42,43,44]. Mechanistically, *M. persicae* possesses a highly evolved xenobiotic metabolism system characterized by the constitutive overexpression of key detoxification enzymes, including cytochrome P450 monooxygenases, esterases, and glutathione S-transferases (GSTs) [45]. These enzymes biotransform plant secondary metabolites (e.g., furanocoumarins and alkaloids) into inactive, excretable forms. Simultaneously, *M. persicae* employs a behavioral mitigation strategy by precisely navigating its stylets into the phloem sap, which contains lower concentrations of toxic compounds than surrounding tissues [44]. This phloem-feeding behavior, combined with a high rate of sap ingestion and rapid honeydew excretion (“flushing”), minimizes the systemic accumulation and toxic effects of ingested defensive compounds, thereby effectively neutralizing the plant’s chemical defenses.

Thus, a clearer understanding of the mechanisms underlying these aphid infestations will require future studies. These should investigate key areas such as the aphid dynamics across seasons and landscapes; the identification of spider and parasitoid species interacting with *M. persicae* in *Ruta* systems; the influence of agronomic practices and neighboring vegetation; and how variability in plant defense chemistry modulates host susceptibility. Such integrated studies will clarify whether *R. chalepensis* serves as an occasional host or a potentially significant reservoir for *M. persicae*.

## 5. Conclusions

This study reports a novel pest–host interaction between aphids (e.g., *M. persicae*) and *R. chalepensis*, with potential implications for integrated pest management. Although we did not investigate the chemical ecology underlying host susceptibility in depth, our observations indicate that even chemically defended plants can become vulnerable to generalist aphids under monoculture and managed conditions. Whether *R. chalepensis* serves only as an occasional host or as a more consistent reservoir for *M. persicae* remains an open question that warrants further investigation.

## Data Availability

All the data is contained in the article and can be made available in the event of requests.
